# CMOS-NEMS Copper Switches Monolithically Integrated Using a 65 nm CMOS Technology

**DOI:** 10.3390/mi7020030

**Published:** 2016-02-15

**Authors:** Jose Luis Muñoz-Gamarra, Arantxa Uranga, Nuria Barniol

**Affiliations:** Department of Electronics Engineering, Universitat Autònoma de Barcelona (UAB), Barcelona 08193, Spain; jose-luis.munozgamarra@cea.fr (J.L.M.-G.); arantxa.uranga@uab.es (A.U.)

**Keywords:** CMOS-NEMS, NEMS, NEMS switch, copper switch

## Abstract

This work demonstrates the feasibility to obtain copper nanoelectromechanical (NEMS) relays using a commercial complementary metal oxide semiconductor (CMOS) technology (ST 65 nm) following an intra CMOS-MEMS approach. We report experimental demonstration of contact-mode nano-electromechanical switches obtaining low operating voltage (5.5 V), good *I*_ON_/*I*_OFF_ (10^3^) ratio, abrupt subthreshold swing (4.3 mV/decade) and minimum dimensions (3.50 μm × 100 nm × 180 nm, and gap of 100 nm). With these dimensions, the operable Cell area of the switch will be 3.5 μm (length) × 0.2 μm (100 nm width + 100 nm gap) = 0.7 μm^2^ which is the smallest reported one using a top-down fabrication approach.

## 1. Introduction

Mechanical switches have emerged as a solution to the increasing static power consumption that metal-oxide-seminconductor field effect (MOSFET) transistors present as their dimensions are reduced [1,2,3]. This problem is solved by the ideally zero leakage power in the OFF state that microelectromechanical (MEMS) switches have, thanks to the air gap defined between the mechanical structure and driver. Moreover, mechanical switches break some of the limits that switches based on transistors present as subthreshold swing (0.1 mV/decade [4,5]) and *I*_ON_/*I*_OFF_ ratio (10^11^ [4]). As their dimensions are reduced faster time response (nano seconds regime [6,7]) are obtained and its integration density is increased. In addition they can be operated in harsh environment [8].

However, there are several challenges that need to be overcome for a large scale production [9]: lower operating voltages, better reliability, stable contact resistance value, reduction of the adhesive forces and miniaturization to the nanoscale following an easy and reproducible fabrication process. Miniaturization of MEMS structures allows a higher integration density, faster responses and low operating voltages. Mechanical switches in the nanoscale range have been obtained at an expense of non-reproducible bottom-up approaches [6] or using dedicated and difficult top-down fabrication processes [10,11]. On the other hand, CMOS-NEMS devices are presented as a promising candidate as its fabrication process takes benefits of the robustness and reproducibility that commercial CMOS technologies present. Some works have appeared that develop and study MEMS switches built using the back end of line (BEOL) layers of commercial CMOS technologies [12,13,14,15], define MEMS structures after the CMOS fabrication [16,17,18] or are based on a CMOS compatible material with additional processes to define the structure or improve the contact [7,19,20].

Nevertheless, further efforts are required to increase the miniaturization of the fabricated structures following an intra CMOS-MEMS approach that allows the monolithic integration of these structures with CMOS circuitry without any additional process.

In order to keep on profiting the advantages of a CMOS fabrication process and decrease the dimensions of the built structures we have chosen a small technology node (ST Microelectronics 65 nm [21]) to develop a nanoelectromechanical (NEMS) switch using back-end-of line (BEOL) metals, based on copper.

## 2. CMOS-NEMS Switch Device Design

**Figure 1:**
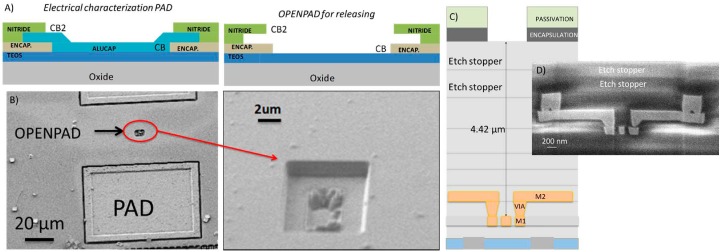
(**A**) Schematic of the electrical pad (PAD in figure) and open pad (OPENPAD in figure) configuration. (**B**) Scanning electronic microscope (SEM) image of an electrical PAD and OPENPAD (with additional zoomed SEM image) before the releasing process. (**C**) Schematic cross section of the CMOS technology showing OPENPAD, etch stoppers layers in the BEOL metal layers and M1 NEMS structure with M2 anchors before the releasing process. (**D**) SEM image of a field ion beam cross section of a M1-M2 NEMS structure before the releasing process.

We have extrapolated the technological approach, previously used in AMS 0.35 μm [22] and UMC 0.18 μm [23] to ST 65 nm CMOS technology [24] where sub-100 nm dimensions can be defined. The NEMS devices are designed along the CMOS process, using the back-end-of-line metal 1 as structural layer and the silicon oxide surrounding the structure as the sacrificial layer. Once the chip is received from the CMOS foundry, a post-CMOS etching process allows the releasing of the final NEMS resonator [24]. The etching process is realized without the addition of any protection mask. In fact in the CMOS design one design rule is violated: the aperture in nitride and encapsulation layers (CB and CB2 in Figure 1A), traditionally performed in order to define the electrical pads, are not filled by the metal, allowing the etchants to have a direct path to reach the sacrificial oxide. Using a combination of dry etching (to overcome the etch stoppers layers used in this Cu based CMOS technology, see Figure 1C), and buffered HF bath for the silicon oxide etching, all the oxide on top of the structure (4.4 μm in M1 switches, Figure 1C), and partially the oxide under it for NEMS releasing, is erased. Nitride and Encapsulation layers protect the area of the chip that does not need to be released (*i.e.*, signal processing circuitry and NEMS anchors).

**Figure 2:**
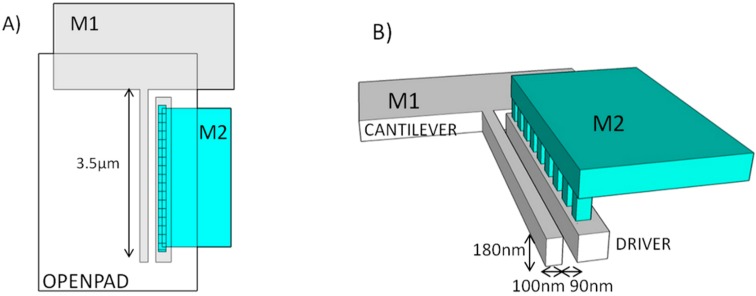
Schematic view of M1 configuration in order to get a 90 nm gap. (**A**) Top view schematic, showing M1 and M2 layers and open pad for releasing. (**B**) Three dimensional schematic showing M2 layer and M2-M1 vias to anchore the M1 driver and keep a reduced 90 nm gap between cantilever and driver.

**Figure 3:**
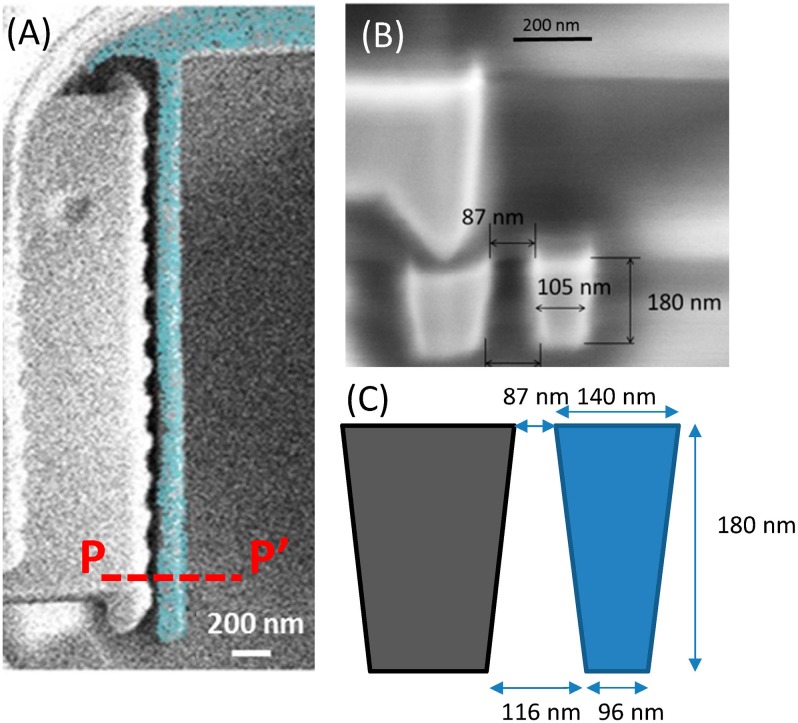
(**A**) Released 2T M1 switch (*l* = 3.5 μm) (Cu cantilevered switch has been colored for easy recognition). (**B**) SEM image of a field ion beam cut (P–P´) before the releasing process (**C**) Schematic view of the switch cross-section with dimensions.

In ST 65 nm commercial CMOS technology, the gap between two layout polygons in a given layer depends on the length in parallel between them and on their width. So in order to be able to define the smallest gap (90 nm) a minimum driver width (width < 200 nm) is defined in metal 1 layer and it is anchored using M2 layer (Figure 2). In Figure 3, the SEM image of a two terminal (2T) switch developed using Metal 1 is presented. It can be observed in the field ion beam cut (Figure 3B) how the beam presents a trapezoidal cross section. This fact will have to be taken into account in order to determine its snap-in voltage. The moment of inertia for a trapezoidal cross section beam is given by expression [25]:

(1) $$I=\frac{1}{48}\left(b_{1}+b_{2}\right)\cdot \left(b_{1}^{2}+b_{2}^{2}\right)\cdot t$$

where *b*_1_, *b*_2_ are the width of the top and bottom side (*b*_1_ = 140 nm and *b*_2_ = 96 nm) and *t* is the thickness of the beam (*t* = 180 nm). Therefore, the spring constant of the cantilever can be calculated using equation [25] $k=3EI/l^{3}$, where *E* is the copper Young’s modulus (*E* = 117 GPa [26]) and *l* the length of the cantilever (*l* = 3.5 μm). The spring constant has a value of 0.2 N/m, almost twice the value of the spring constant assuming a simple square cross section with a width of 100 nm (0.122 N/m). Additionally, the gap, *s*, is not constant along the beam thickness. It has a minimum value of 87 nm in the top side and 116 nm at the bottom. So upper and lower bounds can be fixed for the pull-in voltage using these values and Equation (2) [27]:

(2) $$V_{\mathrm{PI}}=\sqrt{\frac{0.88s^{2}k}{C_{o}}}$$

where *C*_o_ = ε*lt*/*s* (ε = 8.85 × 10^−12^ F/m). So, theoretically the beam should collapse with the electrode when the voltage difference reach a value ranged from 4.7 to 7.3 V. From Equations (1) and (2) it can be observed how the miniaturization of the structures (with lower width and gap) is translated into lower operating voltages.

## 3. Results and Discussion

The 2T switches were characterized using the parametric semiconductor analyzer B1500A from Agilent (Santa Clara, CA, USA). A voltage sweep is applied on the electrode while the beam is polarized to GND. The current is measured in the two terminals. In addition a 25 MΩ resistance was connected in series with the cantilever to prevent its damage during hot switching.

**Figure 4:**
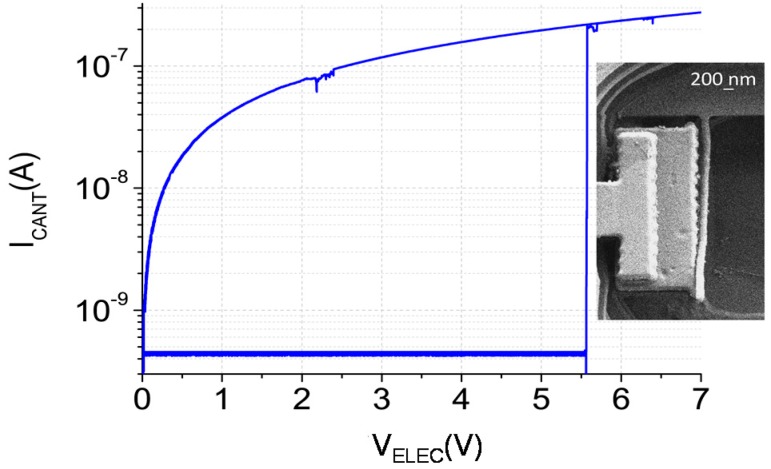
Switch electrical response with a protection resistance of 25 MΩ (Inset SEM image after I/V curve).

**Figure 5:**
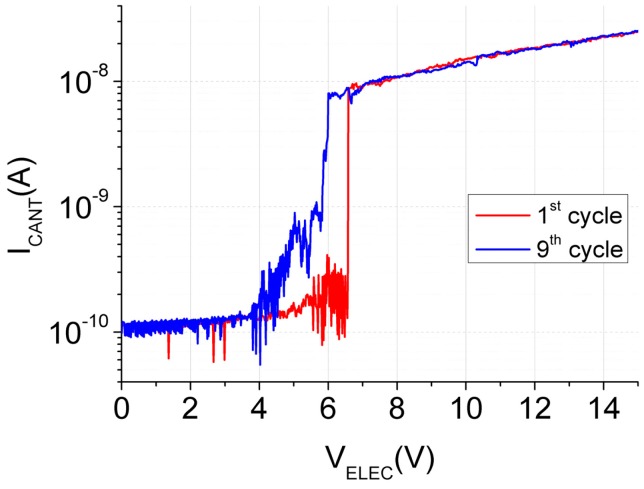
Switch electrical characterization in different successive cycles.

Figure 4 shows the electrical response of the Cu CMOS-NEMS switch performed in ambient conditions. Snap-in event takes place at 5.5 V, inside the theoretical range previously fixed. An *I*_ON_/*I*_OFF_ ratio of 10^3^ was measured with a subthreshold swing of 4.3 mV/decade, beating the MOSFET limit. However, the switch just worked for one cycle and remained in contact with the electrode, probably due to irreversible damage by microwelding. To protect the device from high current density in the small contact area, which can produce microwelding, the value of the protection resistance was increased to 500 MΩ. The electrical characterization of a new device using this protection resistance is shown in Figure 5 for different successive cycles. It can be observed how the snap-in voltage is bigger in this device, but still in the interval fixed theoretically. This pull-in shift could be caused by a slight variation of the device dimensions (metal layer dimensional tolerance).

According to the electrical characterization in Figure 5, the CMOS-NEMS copper switch shows a snap-in event at 6.5 V. An *I*_ON_/*I*_OFF_ ratio of 10^2^ and abrupt transition below 10 mV/decade between the ON and the OFF state is also demonstrated. Note how the subthreshold swing is reduced due to a lower value of *I*_ON_ current limited by the used protection resistance, which at the same time, improves the reliability of the device avoiding melting. The snap-in event varies slightly in the different cycles and its response degrades a bit as long as the cycles are performed. In ambient conditions, the operation of the switch is degraded in two different aspects, due to the native oxide grown on the Cu surface of the switch: (i) charges can be trapped modifying its snap-in voltage [28] and (ii) the contact resistance value will be increased and will make it more unstable. In this case the switch operates for tens of cycles.

**Figure 6:**
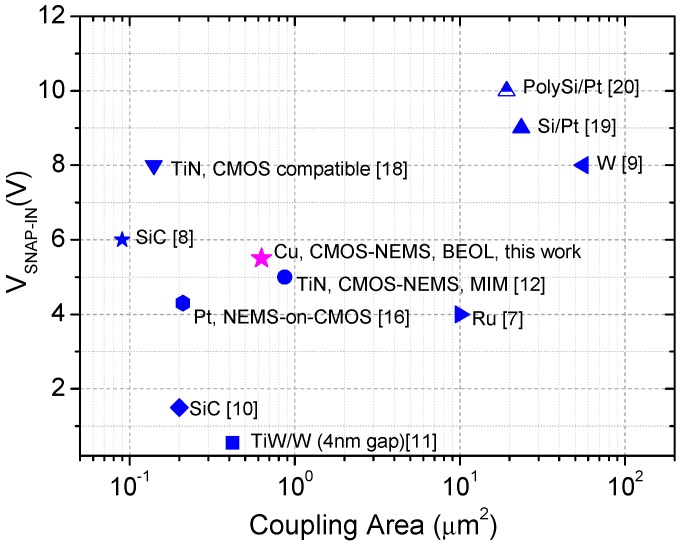
Top-down mechanical switches state of the Art.

Figure 6 summarizes the experimental top-down mechanical switches already reported showing its coupling area and snap-in value. This level of miniaturization and performance has been only surpassed by other less efficient manufacturing methods in terms of production costs [10,11,16]. Although hybrid CMOS/BEOL-NEMS technology has also been announced as candidate for low power and very low cell-footprint [14,15], experimental prototypes are still not reported. These works propose to profit from the air-gap inside the deep submicron CMOS technology (<14 nm) to define vertical three dimensional relays using the BEOL layers with very low elastic constant due to the vertical alignment and keeping the active footprint area of the electrical contact very small. However, in these papers only experimental relays using no-BEOL-CMOS technology are reported. In our case we are reporting experimental evidence of minute relays already fabricated in a commercial CMOS technology (65 nm). Finally and in comparison with [12], in which a CMOS-MEMS approach based on metal-insulator-metal structures is used for the definition of out-of-plane switches, two main benefits from the Cu BEOL approach are achieved with the presented device: (i) higher miniaturization levels up to a 27% coupling area reduction; (ii) the use of an in-plane configuration allows the definition of three terminal devices as the gaps are defined by lithography, as opposed to a deposited sacrificial layer thickness in the out-of-plane configuration used in [12]. Additionally, the in-plane configuration is more robust in front of mechanical stress of the structural cantilever which directly changes the actuation gap and consequently the prediction of the pull-in voltages in the out-of-plane configuration. In order to enhance the device reliability, the contact could be improved by coating the devices with an additional layer, that will be used as the contact material [29,30] or with a better ambient conditions control thanks to an hermetic sealing packaged [31]. Moreover, it has been demonstrated [32] that a Ruthenium liner can be used in a smaller technology node (10 nm) whose BEOL are based on Cu. It will ensure a higher miniaturization level of Cu structures and an improvement of the contact thanks to Ru properties: high hardness, high melting point and conductive oxide.

## 4. Conclusions

Low operating voltage (5.5 V) nanoelectromechanical switches have been fabricated following a CMOS-NEMS approach using the BEOL copper layers of a commercial 65 nm CMOS technology. Good *I*_ON_/*I*_OFF_ ratio (10^3^) and abrupt sub-threshold swing (4.3 mV/decade) were observed with a high miniaturization level (coupling area below 1 μm^2^) beating other top-down fabrication processes that are less efficient and more complicated.
